# A Novel Disease (Water Bubble Disease) of the Giant Freshwater Prawn *Macrobrachium rosenbergii* Caused by *Citrobacter freundii*: Antibiotic Treatment and Effects on the Antioxidant Enzyme Activity and Immune Responses

**DOI:** 10.3390/antiox11081491

**Published:** 2022-07-29

**Authors:** Caiyuan Zhao, Huagen Wen, Shengsheng Huang, Shaoping Weng, Jianguo He

**Affiliations:** 1 State Key Laboratory for Biocontrol, School of Marine Sciences, Sun Yat-sen University, No.132 Waihuan Dong Road, Higher Education Mega Center, Guangzhou 510006, China; zhaocy5@mail.sysu.edu.cn; 2 Southtern Marine Science and Engineering Guangdong Laboratory (Zhuhai), School of Life Science, Sun Yat-sen University, No.132 Waihuan Dong Road, Higher Education Mega Center, Guangzhou 510006, China; wenhg3@mail2.sysu.edu.cn (H.W.); huangshsh29@mail2.sysu.edu.cn (S.H.); lsswsp@mail.sysu.edu.cn (S.W.)

**Keywords:** *Macrobrachium rosenbergii*, water bubble disease, *Citrobacter freundii*, antibiotic treatment, antioxidant enzyme activity

## Abstract

The giant freshwater prawn, *Macrobrachium rosenbergii*, is an important and economical aquaculture species widely farmed in tropical and subtropical areas of the world. A new disease, “water bubble disease (WBD)”, has emerged and resulted in a large loss of *M. rosenbergii* cultured in China. A water bubble with a diameter of about 7 mm under the carapace represents the main clinical sign of diseased prawns. In the present study, *Citrobacter freundii* was isolated and identified from the water bubble. The optimum temperature, pH, and salinity of the *C. freundii* were 32 °C, 6, and 1%, respectively. A challenging experiment showed that *C. freundii* caused the same typical signs of WBD in prawns. Median lethal dose of the *C. freundii* to prawn was 10^4.94^ CFU/g. According to the antibiogram tests of *C. freundii*, florfenicol and ofloxacin were selected to evaluate their therapeutic effects against *C. freundii* in prawn. After the challenge with *C. freundii*, 86.67% and 72.22% survival of protective effects against *C. freundii* were evaluated in the oral florfenicol pellets and oral ofloxacin pellets feding prawns, respectively, whereas the mortality of prawns without fed antibiotics was 93%. After antibiotic treatment and *C. freundii* infection, the activities of superoxide dismutase (SOD), catalase (CAT), glutathione peroxidase (GPx), glutathione S-transferase (GST), malondialdehyde (MDA), acid phosphatase (ACP), alkaline phosphatase (ALP), and lysozyme (LZM) in the hemolymph and hepatopancreas of the prawns and the immune-related gene expression levels of Cu/Zn-SOD, CAT, GPx, GST, LZM, ACP, anti-lipopolysaccharide factor, crustin, cyclophilin A, and C-type lectin in hepatopancreas were all significantly changed, indicating that innate immune responses were induced by *C. freundii*. These results can be beneficial for the prevention and control of *C. freundii* in prawns.

## 1. Introduction

The giant freshwater prawn, *Macrobrachium rosenbergii*, is a member of arthropods, belonging to genus *Macrobrachium*, which is the largest freshwater and the most favored prawn for aquaculture in tropical and subtropical areas of the world [1,2,3], with annual yields of 290,708 metric tons [4]. *M. rosenbergii* has already been developed into an important economically aquaculture species in China [5,6,7,8]. The outbreaks of bacterial diseases in *M. rosenbergii* aquaculture have been reported, such as muscle necrosis disease caused by *Enterococcus* or *Pseudomonas aeruginosa* [9,10,11], *Aeromonas* species (*A. veronii* and *A. caviae*) and *Vibrio* species (*V. alginolyticus* and *V. parahaemolyticus*) [12,13] caused high-mortality diseases. A new epidemic disease, referred to as “water bubble disease” (WBD), recently occurred in cultured *M. rosenbergii* in China, and it caused a mortality of over 30% in diseased prawns.

As *M. rosenbergii* is an invertebrate, it lacks the typical acquired immunity of vertebrates and entirely relies on its innate immunity system to resist infection by various pathogens [14]. Enhanced knowledge about the immune system of *M. rosenbergii* is crucial for disease management [15]. The innate immune system provides defense against invading bacteria through AMPs, enzymes, and cellular components [16]. Antiproteases, myeloperoxidase, and lysozyme (LZM) have been used to learn about and determine the innate immune status of an organism [17]. During pathogen invasion, host cells produce abundant reactive oxygen species (ROS) to kill the invading pathogens. However, a considerably high level of ROS in the body will cause the destruction and damage of DNA and other biological macromolecules [18]. Several major antioxidant enzymes include glutathione peroxidase (GPx), glutathione S-transferase (GST), catalase (CAT), malondialdehyde (MDA), and superoxide dismutase (SOD), which play important roles in anti-oxidization damage and related coping mechanisms [19,20]. Acid phosphatase (ACP), and alkaline phosphatase (ALP) are symbols of macrophage activation, important components of the lysosome system, and play important roles in the innate immune system by engulfing antigens and LZM intracellularly [21]. C-Type lectin (CTL), cyclophilin A (CypA), and AMPs, such as anti-lipopolysaccharide factor (ALF) and crustins, play important roles in the innate immunity for the recognition of effector molecules and clearance of bacteria, fungi, and viruses [22,23,24,25,26,27,28,29]. Antibiotics are commonly used in aquaculture to control the diseases caused by bacteria [30]. Antibiotics at therapeutic levels are mainly administered for short periods of time via the oral route (the most common route for the delivery occurs by mixing the antibiotic with commercial feed) to groups of prawn. Antibiotics legally used in aquaculture must be authorized by Food and Drug Administration (FDA), and abide by the rules for antibiotic use, comprising permissible routes of delivery, dose forms, withdrawal times, tolerances, and dose rates and limitations of specific species. In Norway, antibiotics were sold in pharmacies or in feed plants approved by the Norwegian Medicines Agency, and the amount of antibiotics used and retain records in prescriptions of veterinarian were mandatory to report [31].

In the present study, we aimed to isolate and identify the pathogen associated with WBD in *M. rosenbergii* using challenge experiments, molecular methods and antibiotic treatment. The activities of antioxidant enzymes (i.e., SOD, CAT, GPx, GST, and MDA), ACP, ALP, and LZM and the immune-related gene expression levels of Cu/Zn-SOD, CAT, GPx, GST, LZM, ACP, ALF, crustin, CypA, and CTL in *M. rosenbergii* after *C. freundii* challenge were investigated. The results of the present study will expound the pathogenesis of bacteria in *M. rosenbergii* and facilitate the further prevention and control of *C. freundii* in *M. rosenbergii*.

## 2. Materials and Methods

### 2.1. Strain Isolation and Identification

In November 2019, 30 *M. rosenbergii* (mean body weight 13.5 ± 2.3 g, body length 13.1 ± 1.2 cm) with WBD were collected in Zhaoqing, Guangdong, China. The water bubble diseased *M. rosenbergii* were dissected aseptically, and liquid sample of the water bubble was streaked onto a Luria-Bertani (LB) agarose plate (tryptone 10 g/L, yeast extract 5 g/L, NaCl 10 g/L, agarose powder 15 g/L, pH 7.4) (Sangon Biotech, Shanghai, China). The inoculated plates were incubated for 48 h at 28 °C, and single colonies were obtained and labeled GDZQ201912. After incubation in liquid LB culture (tryptone 10 g/L, yeast extract 5 g/L, NaCl 10 g/L, pH 7.4) for 24 h, the bacteria were collected and stored at −80 °C in 50% glycerol (*v*/*v*).

Biochemical characterization of the isolated strain was identified by Gram staining, shape, and motility, followed by biochemical tests and carbohydrate utilization test. The biochemical tests were performed with GEN III MicroPlate on BIOLOGY (Biology, Vacaville, CA, USA). Transmission electron microscopy (TEM) (JEOL JEM-1400, JEOL, Tokyo, Japan) was used to observe the isolated strain at 120 kV.

### 2.2. Gene Sequencing and Phylogenetic Analysis

Genomic DNA of the bacterial isolate was extracted by using FastPure Cell/Tissue DNA Isolation Mini Kit (Vazyme, Nanjing, China). The 16S rRNA gene of the bacterium was amplified for polymerase chain reaction (PCR) analysis in accordance with the method of Lee et al. [32]. The following amplification primers were used: F (5′-AGAGTTTGATCCTGGCTCAG-3′) and R (5′-CGGTTACCTTGTTACGACTT-3′) (NR_176804.1). The PCR program was carried out as follows: denaturation at 95 °C for 10 min, 30 cycles of denaturation at 95 °C for 30 s, annealing at 53 °C for 30 s, extension at 72 °C for 80 s, and a final extension at 72 °C for 10 min. PCR products were run on a TBE-agarose gel, and the DNA was purified using a TaKaRa MiniBEST Universal Genomic DNA Extraction Kit (Takara, Kusatsu, Japan) and sub-cloned into the pMD19-T Easy Vector (Takara, Japan) for sequencing.

The 1416 bp PCR product was sequenced and blast analyzed. The 16S rRNA gene sequence of GDZQ201912 was aligned with the 16S rRNA gene sequences of other *Citrobacter* (KC210829.1, FN997639.1, MG011554.1, AP022399.1, AP022486.1, and AP022513.1), *Edwardsiella* (FJ405305.1, EF467289.1, GQ180182.1, FJ405309.1, GQ180181.1, and KC309472.1), *Vibrio* (MT071600.1, MT269596.1, MT307282.1, and MT505697.1), and *Aeromonas* species (EU770272.1, KF358430.1, MK182872.1, and MK182893.1) retrieved from the National Center for Biotechnology Information database. The phylogenetic tree based on 16S rRNA gene sequences of the bacteria cells was constructed via the neighbor-joining (NJ) method with the MEGA program (version 7) using a maximum composite likelihood model. The robustness of the NJ tree was assessed using the approximate likelihood-ratio test and bootstrapping with 1000 replicates. Bootstrap values were shown at each node.

### 2.3. Effects of Temperature, pH, and Salinity on the Growth of Citrobacter freundii

The isolated strain was inoculated into the LB liquid medium and homogenized. Different temperatures (24 °C, 28 °C, 32 °C, and 42 °C) were used to test their effects on the growth of *C. freundii* at pH 7.0 and salinity 1%. Different pH (3.0, 4.0, 5.0, 6.0, 7.0, 8.0, 9.0, and 10.0) were used to test their effects on the growth of *C. freundii* at 28 °C temperature and 1% salinity. Different salinities (0%, 1%, 2.0%, 3%, 4%, 5%, 6%, and 7%) were used to test their effects on the growth of *C. freundii* at 28 °C temperature and pH 7.0. A 210 µL LB liquid medium containing the isolated strain (10^2^ CFU/mL) of different temperatures, pH, and salinities was added to each well of 96-well plates. Then, the bacteria were sampled regularly in triplicate to determine the optical density at a wavelength of 600 nm (OD_600_) per 30 min and cultured for 24 h.

### 2.4. Antibiotic Susceptibility Test

The antibiotic susceptibility test was performed using 10^6^ CFU/mL isolated strain in the disk diffusion method as described by Lalitha [33]. The isolated strain was tested following the antimicrobial agents (disk content indicated in parentheses): penicillin (10 µg), doxycycline (30 µg), florfenicol (30 µg), norfloxacin (10 µg), ofloxacin (5 µg), amoxicillin (20 µg), ampicillin (10 µg), cefepime (30 µg), ceftriaxone (30 µg), neomycin (30 µg), gentamicin (10 µg), streptomycin (10 µg), clindamycin (2 µg), lincomycin (2 µg), tetracycline (30 µg), novobiocin (30 µg), polymyxin B (300 µg), and rifampin (5 µg) (HANGWEI, Beijing, China). The sizes of the inhibition zones were measured and recorded triplicately. The experimental results were judged in accordance with the clinical and laboratory standards institute standards [34].

### 2.5. Experimental Challenge

Prawns were obtained from an aquaculture farm in Guandong and acclimated in a recirculating-water aquarium system filled with aerated freshwater for a week. The prawns were fed twice per day with commercial pellet feed (Table 1). Water was maintained at 28 ± 1 °C using a water heater. Healthy prawns (11.00 ± 1.38 g) were used in the following challenge tests. The challenge tests included two groups: a control group, in which 30 prawns were injected intramuscularly with 100 µL phosphate-buffered saline (PBS) at the junction between the 3rd and 4th abdominal segments, and an infected group, in which 30 prawns were injected with 100 µL bacteria inocula (10^6.76^ CFU/g) at the same junction. The 30 prawns of the normal or infected group were stocked in three 36 L aquaria (10 animals per aquarium). The survival rates and clinical signs of the infected and control groups were recorded daily.

### 2.6. Median Lethal Dose Determination

In total, 240 healthy prawns (8.00 ± 0.79 g) were used and stocked in 36 L aquaria (10 animals per aquarium). The *C. freundii* (10^8.01^ cells/g) were used for the challenge experiments. For each trial, 10 animals were injected intramuscularly with the *C. freundii* (100 µL) at 10-fold serial dilutions (10^0^–10^−6^). Animals serving as negative controls were injected with the same volume of PBS. Each treatment was carried out in triplicate. The inoculum and PBS were injected at the junction between the third and fourth abdominal segments. Dead, moribund animals, and clinical signs in each treatment group were recorded and observed at 24 h intervals and examined by the method described in Section 2.1 to ensure the accuracy of the median lethal dose (LD_50_). The LD_50_ was calculated using the Behrens-Kärber method [35].

### 2.7. Antibiotic Treatment

Based on the results of drug sensitivity tests on GDZQ201912, florfenicol and ofloxacin were selected for the evaluation of their therapeutic effects against *C. freundii* in *M. rosenbergii* by common oral pellets containing antibiotics. Florfenicol and ofloxacin were mixed in the pellets based on the 20.0 mg/kg prawn body weight dosage. All of the pellets were coated on the surface feed using edible oil. In total, 360 healthy prawns (7.00 ± 1.03 g) were used and stocked in 36 L aquaria (10 animals per aquarium). The experiment was divided into two groups depending on the different oral antibiotic mixed pellets and time. Group I prawns was treated with antibiotic pellets at the same time with *C. freundii* injection to evaluate their therapeutic effects against *C. freundii*, and group II was treated with antibiotic pellets at the time of typical signs appear after the challenge with *C. freundii* to evaluate the therapeutic effects of the pellets against *C. freundii*. Each group underwent six trials: feeding with common pellets after *C. freundii* injection, feeding with florfenicol mixed pellets after *C. freundii* injection, feeding with ofloxacin mixed pellets after *C. freundii* injection, feeding with common pellets after PBS injection, feeding with florfenicol mixed pellets after PBS injection, and feeding with ofloxacin mixed pellets after PBS injection. Each treatment included 10 prawns and had 3 replicates, and the experimental observation period was 14 days. The concentration of *C. freundii* used to injected prawns was 100-fold LD_50_ of *C. freundii* in *M. rosenbergii* and the same volume described in Section 2.6. The prawns of PBS injection trials were injected with the same volume of PBS. The relative percent survival (RPS) of each trial was counted by the formula: (1% immunized mortality/% control mortality) × 100% [36].

### 2.8. Immune and Anti-Oxidization Parameter Changes in Hemolymph and Hepatopancreas

The hemolymph and hepatopancreas were also collected from group I in the treatment with antibiotics at 1-, 3-, 5-, 7-, 9-, 11-, and 14-days for the following immune enzyme activity assay. The determination of ACP, ALP, CAT, GPx, GST, LZM, MDA, and SOD activities in the hemolymph and hepatopancreas was performed following the protocols of the commercial kits from Nanjing Jiancheng Bioengineering Institute (Nanjing, China). 

### 2.9. Expressions of Immune-Related Genes in Hepatopancreas

The total RNA of hepatopancreas was extracted using a high-purity total RNA Rapid Extraction Kit (Promega, Madison, WI, USA), in accordance with the manufacturer’s protocol. The final RNA was resuspended in 50 mL diethyl pyrocarbonate water and stored at −80 °C. Easy Script One-Step gDNA Removal and cDNA Synthesis SuperMix (AccurateBiology, Changsha, China) were used to synthesize first-strand cDNA following the manufacturer’s instructions. Quantitative PCR (qPCR) was operated on a LightCycler 480 (Roche Applied Science, Basel, Switzerland) with a final 10 µL reaction volume including 0.5 µL of each primer (10 mM), 5 µL SYBR^®^ Green Realtime PCR Master Mix (AccurateBiology, China), 1 µL cDNA, and 3 µL DNase/RNase-free water. The immune-related gene expression levels of ACP, ALF, CAT, crustin, CTL, Cu/Zn-SOD, CypA, GPx, GST, and LZM were evaluated using qPCR to determine the changes after *C. freundii* infection in prawns, with β-actin acting as the house-keeping gene. The PCR cycling conditions and calculation of the relative expression of each gene were performed as described elsewhere [37]. Table 2 shows the primers used to amplify the above genes.

### 2.10. Statistical Analysis

All data were statistically analyzed using SPSS 21.0 software (IBM, New York, NY, USA), and the mean ± standard deviation was determined. *p*-value < 0.05 and <0.01 was considered significant after each measured datum was subjected to Least-significant Difference test. Different graphemes meant significant differences, and the same graphemes meant no significant difference. All columns in this study were drawn with GraphPad Prism 6.0 (San Diego, CA, USA).

## 3. Results

### 3.1. Clinical Symptoms of WBD in M. rosenbergii

**Figure 1:**
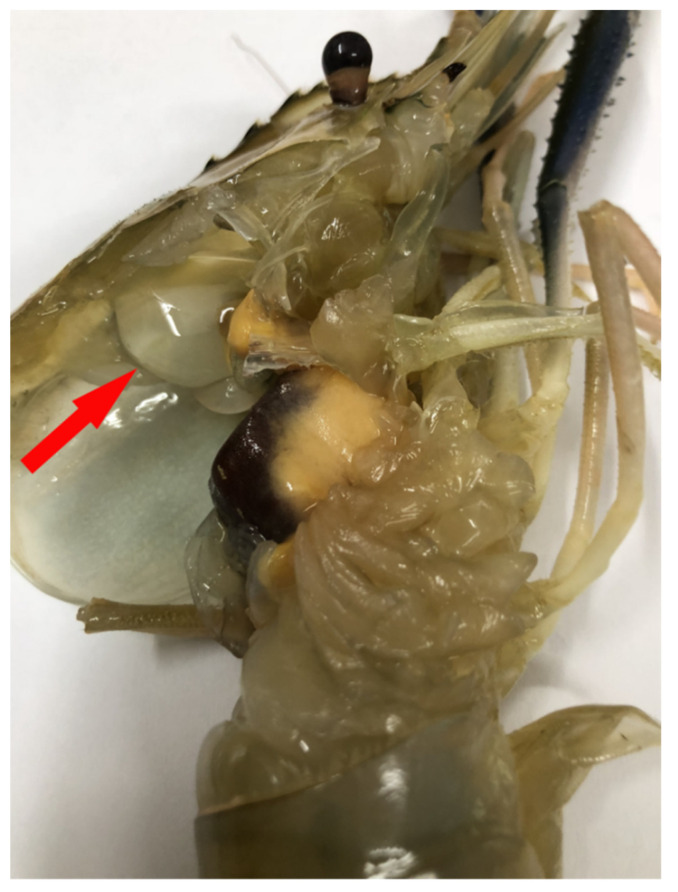
*Macrobrachium rosenbergii* infected with *Citrobacter freundii* showing typical signs of WBD under the carapace (red arrow).

A total of 30 infected *M. rosenbergii* and 30 healthy *M. rosenbergii* were used to examine the clinical signs of *C. freundii* infection. Compared with normal prawns, *C. freundii*-infected prawns showed a 7 mm-diameter bubble under the carapace of the diseased prawn (Figure 1), loss of appetite, inactivity, and weight loss. The 30 infected prawns all had a water bubble under the carapace. *C. freundii* infection caused mass mortality of the infected prawns after the onset of clinical symptoms and the rapid spread of the disease between prawns.

### 3.2. Molecular Identification and Biochemical Characterization

**Figure 2:**
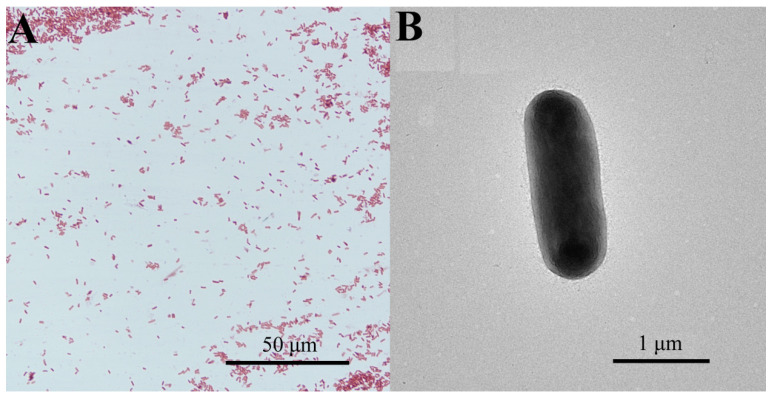
Morphological characteristics and TEM observation of *C. freundii* strain GDZQ201912. (**A**) Gram-stained strain GDZQ201912 observed by light micrograph (1000×). Scale bar 50 µm. (**B**) TEM observation of strain GDZQ201912. Scale bar 1 µm.

The *C. freundii* strain GDZQ201912 was a motile, rod shaped, Gram-negative bacterium (Figure 2A). *C. freundii* was rod-shaped with blunt edges at both ends by TEM (Figure 2B). Ten individuals were randomly measured. The width and length of the bacteria were 0.67–0.76 and 1.88–2.07 µm, respectively.

The 16S rRNA gene amplified from strain GDZQ201912 was sequenced and BLAST analyzed against the non-redundant database, which showed 99.66% identity with *C. freundii* (GenBank Accession Number: CP060662.1). Table 3 and Table 4 present the physiological and biochemical characteristics of strain GDZQ201912, respectively. The molecular sequence and biochemical analyses revealed that the isolated bacterium was *C. freundii*.

### 3.3. Phylogenetic Analysis

**Figure 3:**
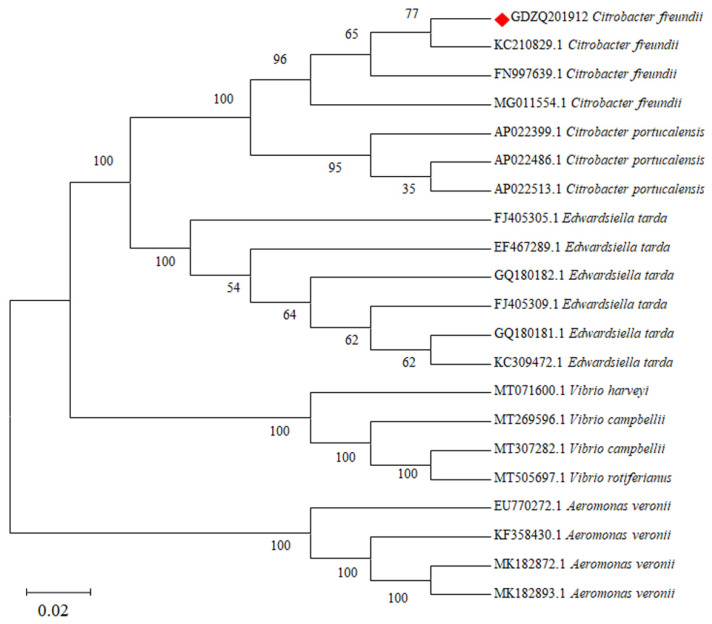
Phylogenetic tree analysis of *Citrobacter* sp. and other pathogenic bacterium species based on 16S rRNA nucleotide sequences. The tree was generated using NJ method by the MEGA 7 software. Bootstrap values above 50% are shown at the nodes. The isolate strain GDZQ201912 identified in this study is indicated by the red shaded diamond.

A phylogenetic tree was constructed using the NJ method by the MEGA 7 software based on the 16S rRNA gene sequences of strain GDZQ201912, and the phylogenetic tree revealed the relationship of strain GDZQ201912 with other *Citrobacter* and pathogenic bacterium species. Bootstrap values above 50% are shown at the nodes. The 16S rRNA gene sequence of strain GDZQ201912 was found to be evolutionarily close to *C. freundii* with the highest bootstrap value (Figure 3).

### 3.4. Effects of Temperature, pH, and Salinity on the Growth of C. freundii

**Figure 4:**
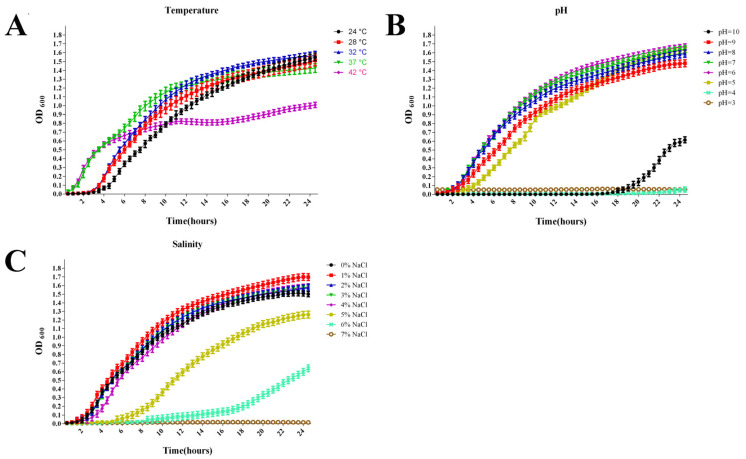
Growth (OD_600_) of strain GDZQ201912 at different temperatures (**A**), pH (**B**), and salinities (**C**).

Figure 4 shows the effects of temperature, pH, and salinity on the growth of *C. freundii*. *C. freundii* exhibits a wide adaptability to temperature. It can grow well in a range from 24 °C to 42 °C (Figure 4A). The optimum temperature was 32 °C. *C. freundii* has a wide pH range and can grow normally at pH 4–9, whose optimal value was around pH 6 (Figure 4B). The maximum salt tolerance of *C. freundii* was 6%, whose optimum value was 1% (Figure 4C). Our results indicated that *C. freundii* has a wide range of temperature, pH, and salinity tolerance.

### 3.5. Experimental Challenge

**Figure 5:**
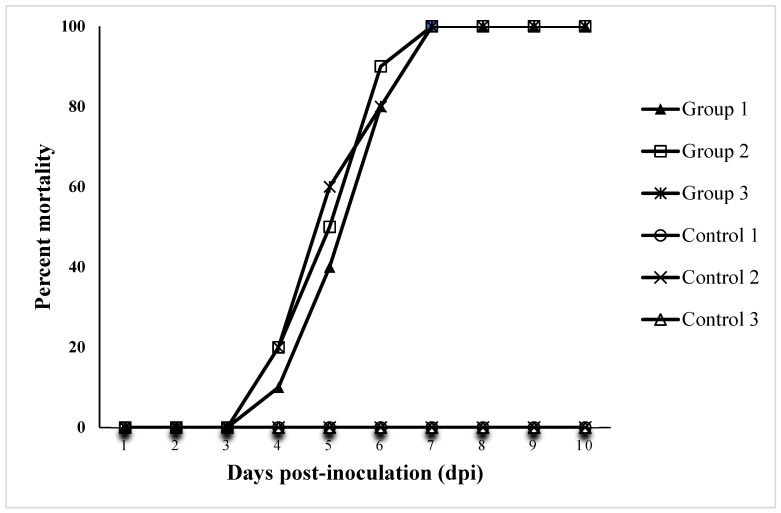
Cumulative mortality curves of the experimentally infected *M. rosenbergii* with strain GDZQ201912. Controls 1, 2, 3, and three replicates of healthy prawns injected with PBS; Groups 1, 2, 3, and three replicates of healthy prawns injected with bacterial inoculum. Each trial contained 10 animals.

Prawns challenged with strain GDZQ201912 died from the third day post-injection (dpi), and no death occurred in the control groups (Figure 5). The challenged prawns exhibited the typical signs of WBD as described previously. The diseased prawns were similar to those observed in naturally infected prawns in 2019. The bacteria re-isolated from the water bubble of challenged prawns and were reconfirmed as *C. freundii*.

### 3.6. LD_50_ of Strain GDZQ201912 in M. rosenbergii

**Figure 6:**
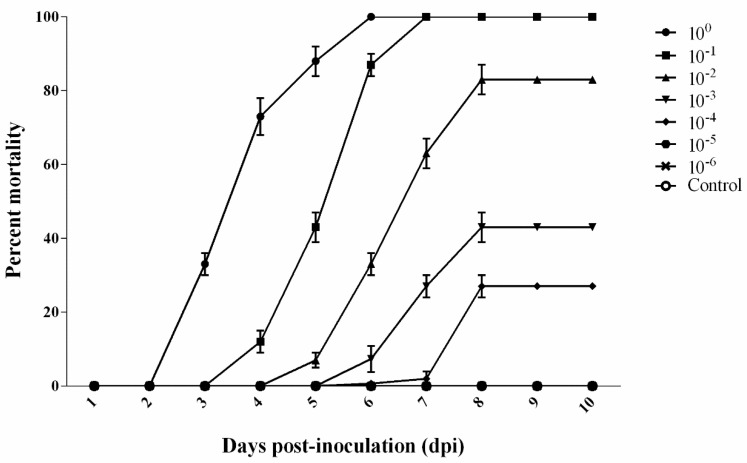
Cumulative mortality curves for the determination of LD_50_ in *M. rosenbergii* challenged with *C. freundii* at different concentrations.

Figure 6 shows the cumulative mortality rate of *M. rosenbergii* after post-infection with *C. freundii*. For each trial, 10 prawns were injected with bacteria inoculum at 10^0^–10^−6^ dilutions, and the control group was injected with same volume of PBS. The control prawns showed no mortality during the experimental challenge. Among *M. rosenbergii* injected with serial dilutions of *C. freundii*, prawns injected with 10^0^, 10^−1^, and 10^−2^ dilutions showed disease signs from 2, 3, and 4 dpi, respectively. Prawns injected with 10^−3^ and 10^−4^ dilutions exhibited disease symptoms from 5 dpi, whereas no death occurred among those injected with 10^−5^ and 10^−6^ dilution during the experimental challenge. The LD_50_ of *C. freundii* was 10^4.94^ CFU/g (Figure 6).

### 3.7. Antibiogram Tests of Strain GDZQ201912

Antibiogram tests of strain GDZQ201912 revealed its resistance to different antibiotics, such as penicillin, ampicillin, clindamycin, lincomycin, and novobiocin, whereas it showed sensitivity to doxycycline, florfenicol, norfloxacin, ofloxacin, amoxicillin, cefepime, ceftriaxone, neomycin, gentamicin, streptomycin, tetracycline, polymyxin B, and rifampin (Table 5). Among the 13 sensitive antibiotics, strain GDZQ201912 was the most sensitive to cefepime, followed ceftriaxone, ofloxacin, and florfenicol in fourth.

### 3.8. Antibiotic Treatment

**Figure 7:**
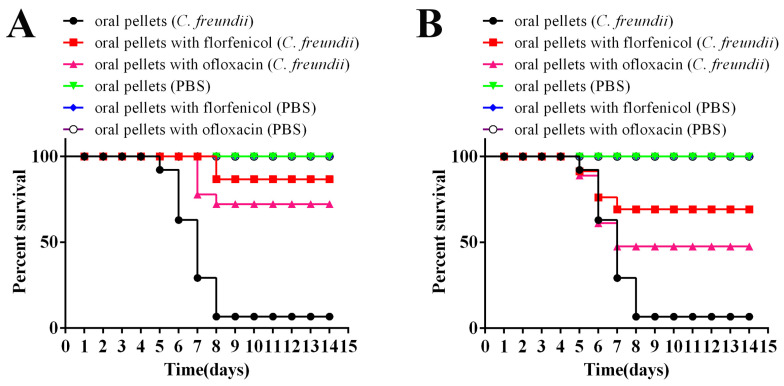
RPS of florfenicol and ofloxacin treatment at different time points after the challenge intramuscular with 100-fold LD_50_ *C. freundii* strain GDZQ201912 for 14 days. (**A**) RPS of prawns treated with antibiotic pellets at the same time with *C. freundii* challenge. (**B**) RPS of prawns treated with antibiotic pellets after death occurred post *C. freundii* challenge.

The *M. rosenbergii* mortalities caused by florfenicol and ofloxacin treatment at different time points after the intramuscular challenge with *C. freundii* or PBS were monitored for 14 days (Figure 7). Prawns fed with common pellets after *C. freundii* infection died from 5 dpi, and the death reached the maximum on 8 dpi with mortalities of 93.0%. In Group I, prawns fed with florfenicol mixed pellets after *C. freundii* infection died from 8 dpi, and the death reached the maximum on 8 dpi with mortalities of 19.40%; prawns fed with ofloxacin mixed pellets after *C. freundii* infection died from 7 dpi, and death reached the maximum on 8 dpi with mortalities of 32.83%. In Group II, prawns fed with florfenicol and ofloxacin mixed pellets after *C. freundii* infection all died from 5 dpi, and the death reached the maximum on 7 dpi with mortalities of 35.67% and 57.47%, respectively. No death was observed among prawns fed with common pellets and mixed pellets of florfenicol and ofloxacin after PBS injection. The evaluation of the therapeutic effects of antibiotic pellets revealed that the RPS of florfenicol and ofloxacin in group I against *C. freundii* in prawns were 86.67% and 72.22%, respectively (Figure 7A). The RPS of florfenicol and ofloxacin in Group II against *C. freundii* in prawns were 69.17% and 47.53%, respectively (Figure 7B). Both florfenicol and ofloxacin initially showed effective protection for *M. rosenbergii* against *C. freundii* (and especially florfenicol).

### 3.9. Immune and Antioxidant Enzyme Activity Changes in Hemolymph

**Figure 8:**
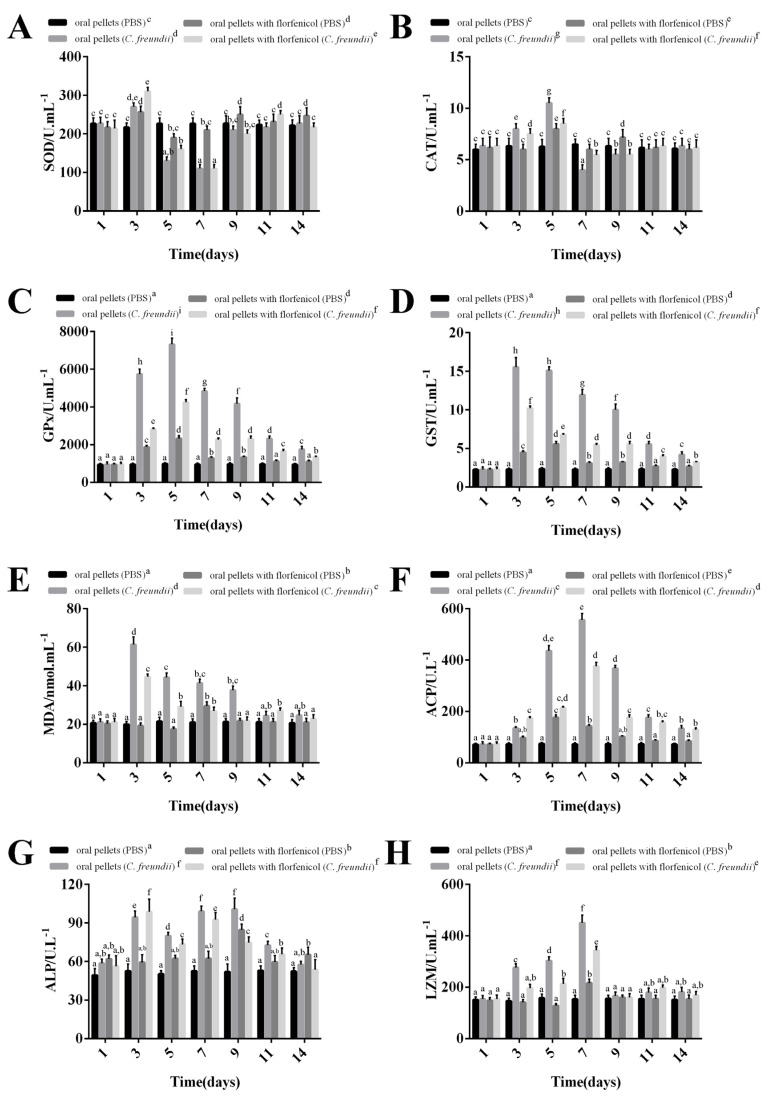
Changes in non-specific immune parameters of *M. rosenbergii* hemolymph at 1-, 3-, 5-, 7-, 9-, 11-, and 14-days after *C. freundii* infection. (**A**) SOD, (**B**) CAT, (**C**) GPx, (**D**) GST, (**E**) MDA, (**F**) ACP, (**G**) ALP, and (**H**) LZM activities. Data are presented as mean ± standard error of the mean (SEM) (*n* = 3); different graphemes (a, b, c, d, e, f, g, h and i) indicate significant difference values of non-specific immune parameters at *p* < 0.05.

The enzymatic activities of SOD, CAT, GPx, GST, MDA, ACP, ALP, and LZM of *M. rosenbergii* were measured at 1-, 3-, 5-, 7-, 9-, 11-, and 14-days after the *C. freundii* strain GDZQ201912 and florfenicol challenge. In hemolymph, the results revealed that the SOD activity in the groups fed with common and florfenicol mixed pellets significantly increased from three days post *C. freundii* infection, rapidly declined at five and seven days (*p* < 0.05, Figure 8A), and increased again to normal levels at 9-, 11-, and 14- days compared with the PBS control (fed with common pellets) group (*p* > 0.05, Figure 8A). No significant differences were observed between the SOD activities in the PBS control (fed with common pellets) groups (*p* > 0.05, Figure 8A).

The CAT activity in *M. rosenbergii* fed with common pellets significantly increased from three-, five-, and nine-days post *C. freundii* infection and reached the peak value at 5 dpi compared with the PBS control (fed with common pellets) group (*p* < 0.05, Figure 8B). This value significantly reduced to the lowest at 7-dpi. During this period, the CAT activity of prawns fed with florfenicol mixed pellets significantly increased from three and five days post *C. freundii* infection and reached the peak value at 5-dpi compared with the PBS control (fed with common pellets) group (*p* < 0.05, Figure 8B). No significant differences were observed between the CAT activities in the PBS control (fed with common pellets) groups (*p* > 0.05, Figure 8B).

The GPx activity in *M. rosenbergii* fed with common pellets significantly increased at 3-, 5-, 7-, 9-, 11-, and 14-days post *C. freundii* infection and reached the peak value at 5-dpi compared with the PBS control (fed with common pellets) group (*p* < 0.01, Figure 8C). During this period, the groups fed with florfenicol mixed pellets (*C. freundii* infection) presented similar trend levels of enzymatic activity, which were lower than that observed for the group fed with common pellets (*C. freundii* infection) and reached the peak value at 5 dpi compared with the PBS control (fed with common pellets) group (*p* < 0.01, Figure 8C). No significant differences were observed between the GPx activities in the PBS control (fed with common pellets) groups (*p* > 0.05, Figure 8C).

The GST activity of prawns fed with common mixed pellets significantly increased at 3-, 5-, 7-, 9-, 11-, and 14-days post *C. freundii* infection and reached the peak value at 3 dpi compared with the PBS control (fed with common pellets) group (*p* < 0.01, Figure 8D). During this period, the groups fed with florfenicol mixed pellets (*C. freundii* infection) presented similar trend levels of enzymatic activity, which were lower than that observed for the group fed with common pellets (*C. freundii* infection) and reached the peak value at 3-dpi compared with the PBS control (fed with common pellets) group (*p* < 0.01, Figure 8D). No significant differences were observed between the GST activities in the PBS control (fed with common pellets) groups (*p* > 0.05, Figure 8D).

The MDA activity of prawns fed with common mixed pellets significantly increased at three, five, seven, and nine days post *C. freundii* infection and reached the peak value at 3-dpi compared with the PBS control (fed with common pellets) group (*p* < 0.01, Figure 8E). During this period, the groups fed with florfenicol mixed pellets (*C. freundii* infection) presented similar trend levels of enzymatic activity, which were lower than that observed for the group fed with common pellets (*C. freundii* infection) and reached the peak value at 3-dpi, compared with the PBS control (fed with common pellets) group (*p* < 0.05, Figure 8E). No significant differences were observed between the MDA activity in the PBS control (fed with common pellets) groups (*p* > 0.05, Figure 8E).

The ACP activity in *M. rosenbergii* fed with common pellets significantly increased from 3-days post *C. freundii* infection and peaked at 7-dpi (556.34 U/L) compared with the PBS control (fed with common pellets) group (*p* < 0.01, Figure 8F). Subsequently, this value declined at 9-, 11-, and 14-days. During this period, the two groups fed with florfenicol pellets (PBS and *C. freundii* infected) presented similar trend levels of enzymatic activity, which were lower than that observed for the group fed with common pellets and reached the peak values of 177.67 and 376.34 U/L on 5- and 7- dpi, respectively (*p* < 0.01). No significant differences were observed in this parameter in the PBS control (fed with common pellets) group (*p* > 0.05, Figure 8F).

The ALP activity of prawns fed with common and florfenicol mixed pellets significantly increased at three-, five-, seven-, and nine-days after *C. freundii* infection, with peak values of 98.67 and 64.67 U/L, respectively, at three and seven days compared with the PBS control (fed with common pellets) group (*p* < 0.01, Figure 8G). No significant differences were observed between ALP activities in the PBS control (fed with common pellets) group (*p* > 0.05, Figure 8G).

The LZM activity in *M. rosenbergii* fed with common pellets significantly increased from 3-, 5-, 7-, 11-, and 14-days post *C. freundii* infection and peaked 450.00 U/mL at 7 days compared with the PBS control (fed with common pellets) group (*p* < 0.01, Figure 8H). This value declined to normal level at 9-dpi (*p* > 0.05, Figure 8H). During this period, the groups fed with florfenicol mixed pellets (*C. freundii* infection) presented similar trend levels of enzymatic activity, which were lower than that observed for the group fed with common pellets and reached the peak value at seven days (*p* < 0.05, Figure 8H). No significant differences were observed between the LZM activities in the PBS control (fed with common pellets) groups (*p* > 0.05, Figure 8H).

### 3.10. Immune and Antioxidant Enzyme Activity Changes in Hepatopancreas

**Figure 9:**
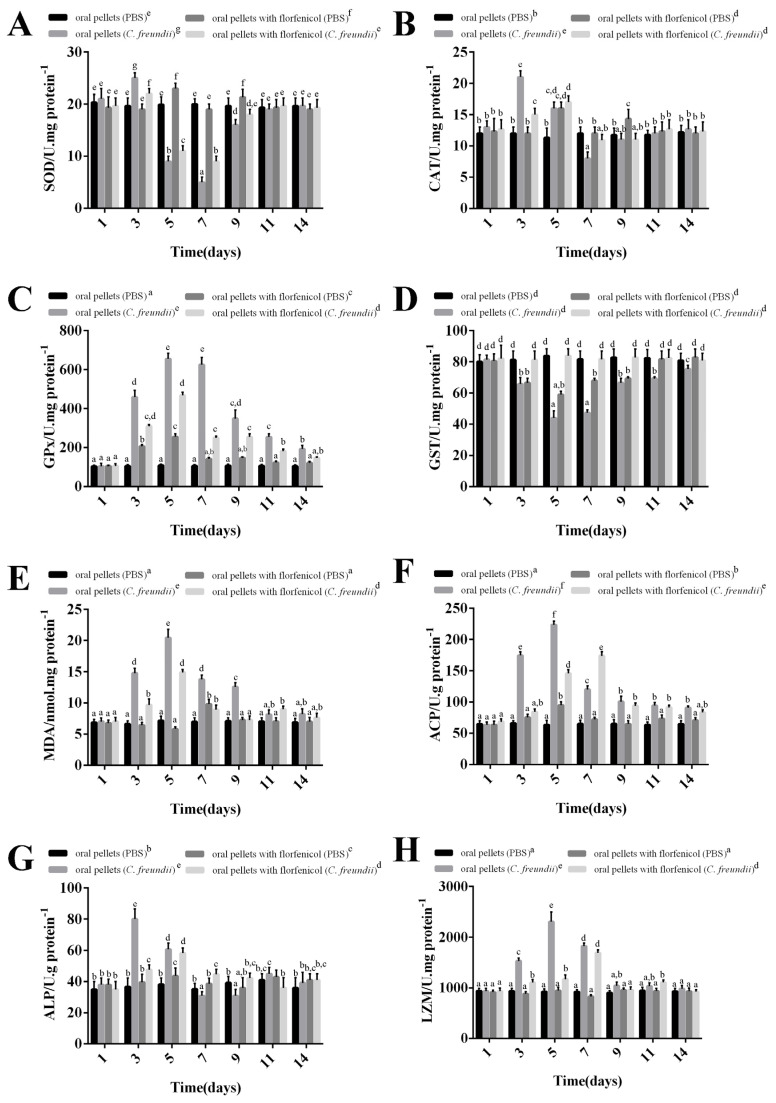
Changes in non-specific immune parameters of *M. rosenbergii* hepatopancreas at 1-, 3-, 5-, 7-, 9-, 11-, and 14-days after *C. freundii* infection. (**A**) SOD, (**B**) CAT, (**C**) GPx, (**D**) GST, (**E**) MDA, (**F**) ACP, (**G**) ALP, and (**H**) LZM activities. Data are presented as mean ± SEM (*n* = 3); different graphemes (a–g) indicate significant difference values of non-specific immune parameters at *p* < 0.05.

The results revealed that the SOD activity in *M. rosenbergii* (fed with common and florfenicol mixed pellets) significantly increased at 3-days, decreased at 5- and 7-days post *C. freundii* infection (*p* < 0.05, Figure 9A), and increased again to normal levels at 11 and 14 days compared with the PBS control (fed with common pellets) group (*p* > 0.05, Figure 9A). No significant differences were observed between the SOD activities in the PBS control (fed with common pellets) groups (*p* > 0.05, Figure 9A).

The CAT activity in *M. rosenbergii* (fed with common and florfenicol mixed pellets) significantly increased at three and five days and reached the peak value at 3- and 5-dpi, respectively, while prawns fed with common pellets decreased at seven days post *C. freundii* injection compared with the PBS control (fed with common pellets) group (*p* < 0.05, Figure 9B). No significant differences were observed between the CAT activities in the PBS control (fed with common pellets) groups (*p* > 0.05, Figure 9B).

The GPx activity in *M. rosenbergii* (fed with common and florfenicol mixed pellets) significantly increased at 3-, 5-, 7-, 9-, and 11-days post *C. freundii* injection and both reached the peak value at 5-dpi compared with the PBS control (fed with common pellets) group (*p* < 0.01, Figure 9C). No significant differences were observed between the GPx activities in the PBS control (fed with common pellets) group (*p* > 0.05, Figure 9C).

The GST activity of prawns (fed with common and florfenicol mixed pellets) significantly decreased at 3-, 5-, 7-, 9-, and 11-days post *C. freundii* injection, compared with the PBS control (fed with common pellets) group (*p* < 0.05, Figure 9D). No significant differences were observed between the GST activities in the PBS control (fed with common pellets) and *C. freundii* infection groups (fed with florfenicol mixed pellets) (*p* > 0.05, Figure 9D).

The MDA activity of prawns (fed with common and florfenicol mixed pellets) significantly increased at 3-, 5-, and 7-days post *C. freundii* injection, compared with the PBS control (fed with common pellets) group (*p* < 0.05, Figure 9E). No significant differences were observed between the MDA activities in the PBS control (fed with common and florfenicol mixed pellets) groups (*p* > 0.05, Figure 9E).

The ACP activity in the hepatopancreas of *M. rosenbergii* fed with common and florfenicol mixed pellets significantly increased at 3-, 5-, 7-, 9-, 11-, and 14-days post *C. freundii* infection and reached the peak value at 5- and 7-dpi, respectively, compared with the PBS control (fed with common pellets) group (*p* < 0.05, Figure 9F). No significant differences were observed between the ACP activities in the PBS control (fed with common pellets and florfenicol mixed pellets) groups (*p* > 0.05, Figure 9F).

The ALP activity of prawns (fed with common and florfenicol mixed pellets) significantly increased at three- and five-days post *C. freundii* injection, compared with the PBS control (fed with common pellets) group (*p* < 0.05, Figure 9G). No significant differences were observed between the ALP activities in the PBS control (fed with common and florfenicol mixed pellets) groups (*p* > 0.05, Figure 9G).

The LZM activity in *M. rosenbergii* fed with common pellets significantly increased at three-, five-, and seven-days post *C. freundii* infection and reached the peak value at 5-dpi compared with the PBS control (fed with common pellets) group (*p* < 0.01, Figure 9H). This value declined to normal levels at 9-, 11-, and 14-dpi. During this period, the groups fed with florfenicol mixed pellets (*C. freundii* infection) presented similar trend levels of enzymatic activity, which were lower than that observed for the prawns fed with common pellets and reached the peak value at 7-days (*p* < 0.05, Figure 9H). No significant differences were observed between the LZM activities in the PBS control (fed with common and florfenicol mixed pellets) groups (*p* > 0.05, Figure 9H).

### 3.11. Expression of Immune-Related Genes in Hepatopancreas after the Challenge with C. freundii

**Figure 10:**
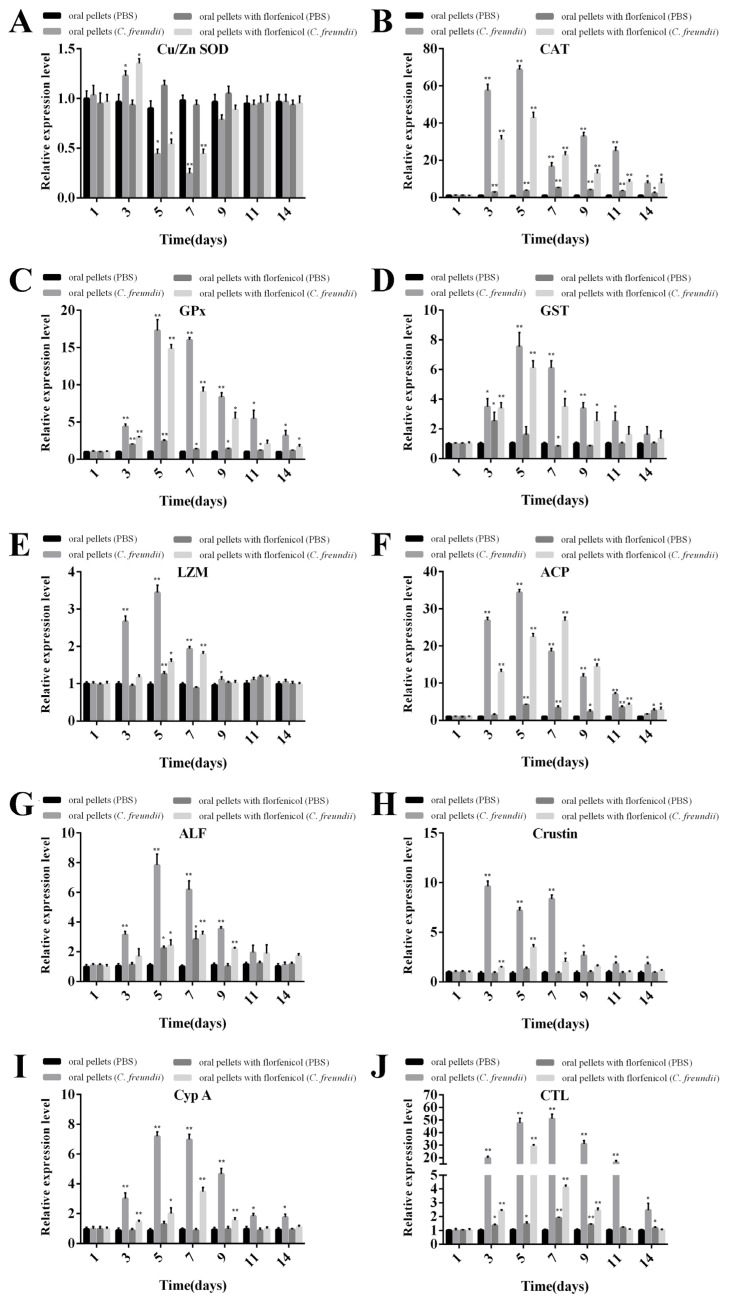
Analysis of immune-related gene expressions in hepatopancreas of *M. rosenbergii* in response to *C. freundii* challenge by RT-PCR at 1-, 3-, 5-, 7-, 9-, 11-, and 14-dpi. (**A**) Cu/ZnSOD, (**B**) CAT, (**C**) GPx, (**D**) GST, (**E**) LZM, (**F**) ACP, (**G**) ALF, (**H**) Crustin, (I) Cyp A, and (J) CTL gene expression levels of immune-related genes were measured by qRT-PCR, and measurement was performed in triplicate for each sample. Expression values were normalized to those of β-actin using the Livak (2^−ΔΔCT^) method, and the expression level detected at 1 day was set as 1.0. Data are presented as mean ± SEM (*n* = 3); * *p* < 0.05, ** *p* < 0.01.

Immunity-related genes (Cu/Zn-SOD, CAT, GPx, GST, LZM, ACP, ALF, crustin, CypA and CTL) mRNA expressions were determined in the hepatopancreas of *M. rosenbergii* at 1-, 3-, 5-, 7-, 9-, 11-, and 14-days after the challenge with *C. freundii* (Figure 10). These genes were up-regulated at different levels following the challenge with *C. freundii* in the groups fed with common and florfenicol mixed pellets compared with the PBS control groups (fed with common and florfenicol mixed pellets). The mRNA expressions of several genes (CAT, GPx, GST, LZM, ACP, ALF, and CypA) in *M. rosenbergii* fed with common pellets reached their peak at five days after the challenged with *C. freundii* (Figure 10B–G,I), whereas the mRNA expressions of crustin (Figure 10H) and CTL (Figure 10J) peaked at 3- and 7-dpi, respectively. The mRNA expressions of several genes (CAT, GPx, GST, crustin and CTL) in *M. rosenbergii* fed with florfenicol mixed pellets reached their peak at 5-dpi (Figure 10B–D,H,J, respectively). Meanwhile, the mRNA expressions of LZM, ACP, ALF and CypA genes peaked at 7-dpi (Figure 10E–G,I, respectively). In addition, the mRNA expression of Cu/ZnSOD in prawns (fed with common and florfenicol mixed pellets) were up-regulated at 3-dpi and down-regulated at 5- and 7-dpi, with the lowest peak value observed at 7-dpi (Figure 10A). No evident difference was reported between the PBS control groups (fed with common pellets).

## 4. Discussion

Diseases are a major constraint in the sustainable development of aquaculture all over the world. The outbreak and spread of bacterial infections in aquaculture farms pose a major concern. *C. freundii* is an opportunistic pathogen that is commonly found in water, soil, animals and has been reported to cause numerous diseases in aquaculture [39,40,41]. In recent years, an increasing number of studies reported *C. freundii* causing diseases and serious mass deaths in various aquatic products, such as Chinese mitten-handed crab [42], red swamp crayfish [14], grass carp [24], and crucian carp [43]. In addition, *C. freundii* can cause infections in amphibians, reptiles, birds, and mammals [36,44,45,46,47,48]. In the present study, *C. freundii* isolated from diseased *M. rosenbergii* was confirmed to be the cause contributing to prawn mortality, with the typical signs of water bubble under the carapace.

Phylogenetic analysis revealed that our isolated strain GDZQ201912 showed a very close association with other strains of *C. freundii* submitted from China and UK and formed separate clades with other *Citrobacter* sp., such as *C. portucalensis* (AP022399.1, AP022486.1, and AP022513.1). The physiological and biochemical profiles of strain GDZQ201912 were similar to those of other isolates reported by earlier works [49,50]. Variations in the biochemical test, such as those of tyrosine, putrescine [38], indole, citrate, H_2_S, malonate, raffinose, and dulcitol, were also described by earlier studies [17,49].

In the case of pathogenic bacteria, such as *C. freundii*, multidrug resistance is one of the most prominent problems [50]. In this study, the isolated strain was resistant to four different β-lactam antibiotics, including ampicillin, clindamycin, lincomycin, and penicillin. Numerous studies demonstrated the existence of β-lactamase in *C. freundii* [51,52,53,54]. The resistant feature against β-lactam antibiotics of our strain indicated the existence of β-lactamase gene, which benefits the strain. The *C. freundii* resistant to tetracycline and oxytetracycline has been isolated from mammals, poikilothermic hosts, poultry, catfish, and rainbow trout [55,56,57,58,59,60].

The innate immune system defense against invading pathogens relies on AMPs, enzymes, and cellular components [16]. During this process, host cells will produce abundant ROS, such as superoxide anion (O_2_^•−^), hydrogen peroxide (H_2_O_2_), hydroxyl radicals (^•^OH), and singlet oxygen (^1^O_2_), and reactive oxygen intermediates to kill the invading pathogens; however, excessive levels of ROS in the body will also cause the destruction and damage of DNA and other biological macromolecules [18]. Crustaceans can rely on complex enzymatic antioxidant systems to alleviate and restore the oxidative damage, SOD, GPx, GST, CAT, MDA, and so on [61]. SODs conduct the first step in eliminating the oxidative damage caused by ROS from cells. The SOD activity first increased at 1.5 h but decreased significantly at 12–48 h in the hepatopancreas of *P. monodon* after *V. parahaemolyticus* infection [62]. In *L. vannamei*, the SOD activity showed remarkable decreases in the hemolymph after 6–96 h and recovery after 96 h post-*V. alginolyticus* injection [63]. The SOD activity was also significantly reduced in *Probopyrus ringueleti*-infected *Palaemontes argentinus* [64]. In addition, the Cu/ZnSOD showed an up-regulated expression in the hemocytes of *M. rosenbergii* at 3 and 6 h after *Lactococcus garvieae* infection, whereas down-regulation was observed in the hepatopancreas at 3 h after *L. garvieae* infection [65]. In *M. nipponense*, the ecCuZnSOD mRNA expression level showed a drastic decrease at 12 h after *A. hydrophila* injection [66]. In *M. rosenbergii*, the cytMn-SOD transcript also decreased in the hepatopancreas at 3 h after *L. garvieae* injection [67]. The expression of icCu/Zn-SOD decreased in the first 8–16 h and then recovered after the challenge with *Listonella anguillarum* and *Micrococcus luteus* in *Chlamys farreri* [68]. The relative expression level of icCu/Zn-SOD mRNA increased rapidly at 6 h post infection with *Vibrio anguillarum* in *Venerupis philippinarum*, followed by a remarkable decrease, and then increased again [69]. In the present study, a similar expression profile was found in the hepatopancreas of *M. rosenbergii* after *C. freundii* infection, in which the relative expression level of Cu/ZnSOD mRNA was up-regulated first and then decrease extremely. Then, the expression level recovered, and SOD activities in hepatopancreas and hemolymph were restored. CAT plays an important role in the antioxidant enzyme defense system to alleviate ROS. The CAT activity in the hepatopancreas of *M. rosenbergii* and CAT mRNA expression increased markedly at 6 and 24 h post injection with *V. parahaemolyticus* [70]. The CAT activity exhibited a significant rise at the early stage of white spot syndrome virus infection in *P. monodon* [71]. The CAT activity in the hepatopancreas of *M. rosenbergii* significantly decreased at 5 days after spiroplasma MR-1008 infection and showed a recovery at 15 days; however, the mRNA expression level was up-regulated and peaked at 12 days [72]. Similar trends were observed in the CAT activity and mRNA expression level in the present study. GST and GPx are involved in different mechanisms for the host defense against oxidative damage [73]. In *Fenneropenaeus chinensis*, the mRNA expression levels of *FcTheta*GST, *FcMu*GST, and *Fc*GPx in the hepatopancreas were up-regulated after the challenge with *V. anguillarum* for 24 h, whereas the GST activity decreased [74]. In *L. vannamei*, the GPx activity and mRNA transcription increased significantly at 12 h after the challenge with *V. anguillarum* [75]. In *P. monodon*, the GPx activity of hepatopancreas increased significantly at 6 h and reached the peak level at 12 h after the *V. parahaemolyticus* challenge; the MDA activity significantly increased from 6 h to 24 h with a peak at 6 h, whereas the GST activity decreased significantly at 3 h [62]. The GPx activity in the gill of *F. chinensis* increased at 6 h and remained at high levels up to 24 h [74]. In addition, the results of increased mRNA expression levels and activities (GPx, GST, CAT, and MDA) during *C. freundii* infection in *M. rosenbergii* were in agreement with the histopathological damage in hepatopancreas.

LZM is an important enzyme of the innate immune defense system against bacterial infection, and it can cause bacterial cell disintegration by hydrolyzing the bacterial cell wall [76,77]. Lysosomal enzymes, which are produced by phagocytes from crayfish *Procambarus clarkii*, can efficiently degrade and eliminate foreign materials, as had been demonstrated by Franchini and Ottaviani [78]. In *P. monodon* and *M. rosenbergii*, the lysosomal activity showed a significant increase after *V. vulnificus* infection and increased in *L. vannamei* infected with *Micrococcus lysodeikticus* [79,80]. ACP and ALP are symbols of macrophage activation, important components of the lysosome system, and play important roles in the innate immune system to engulf antigens intracellularly [21]. The ACP activity increased substantially within 48 h in the hemolymph of *C. farreri* after the treatment with *V. anguillarum* [81]. In *L. vannamei*, the ACP and ALP activities significantly increased with *V. parahaemolyticus* and *M. lysodeikticus* infections, respectively [82]. In addition, the ACP and ALP activities in *M. rosenbergii* were significantly enhanced after the challenge with a novel pathogen spiroplasma MR-1008 [72]. In the present study, the enzyme activities of ACP, ALP, and LZM in the hepatopancreas and hemolymph were all enhanced after *M. rosenbergii* infection with *C. freundii*; the enzyme activities all increased significantly in the hepatopancreas after *T. chinensis* infection in *M. nipponense* [83].

AMPs are the main effector molecules in the innate immunity of crustaceans, and they act as frontline effectors to defend against invading bacteria, fungi, and viruses [26,27,84]. The major AMPs, such as penaeidins, crustins, and ALF, have been identified in shrimp [29]. In *P. monodon*, the mRNA expression level of crustin-like peptide significantly increased at 24 h post-infection with *V. harveyi* and recovered at 72 h [85]. The mortality of crustin-depleted *L. vannamei* by RNA interference (RNAi) had a remarkable increase within 48 h post-infection with *V. penaeicida* [86]. In *Marsupenaeus japonicus*, *Mj*Cru I-1 can enhance the hemocyte phagocytosis, increase shrimp survival rate with *V. anguillarum* and *Staphylococcus aureus* infection, and weaken the bacterial clearance by knockdown of *Mj*Cru I-1 [84]. In *L. vannamei*, *Lv*CrustinB was up-regulated with *V. parahaemolyticus* infection, and the knockdown of *Lv*CrustinB can increase the shrimp mortality rate [87]. The expression level of *Fc*ALF2 in *Fenneropenaeus chinensis* significantly increased after the injection with *M. lysodeikticus* and *V. anguillarum* [88]. The in vivo function of *Lv*ALF1 in response to *V. penaeicida* and *Fusarium oxysporum* infections was measured by RNAi; the *Lv*ALF1-depleted *L. vannamei* caused a significant increase in the mortality of infected shrimps [89]. With *C. freundii* infection, the ALF mRNA expression significantly increased in crayfish *P. clarkii* [14]. The expressions of ALF and crustin increased in the hepatopancreas of *M. rosenbergii* after *C. freundii* infection in the present study; in addition, the ALF expression significantly increased in *L. vannamei* after the *V. anguillarum* and *M. lysodeikticus* challenge [90].

CypA acts as the receptor for the immunosuppressive agent cyclosporin A and belongs to the superfamily of peptidyl-prolyl cis-trans isomerases [91]. The mRNA expression of *bts*CypA was up-regulated in the hepatopancreas of *P. monodon* after stimulation by lipopolysaccharide [92]. After *V. anguillarum* challenge, the expression level of *Cf*CypA was up-regulated in the gonads of *C. farreri* and reached the peak at 4 h post-injection [93]. The mRNA expression level of *Es*CypA increased in the fungal *Pichia pastoris*-infected *E. sinensis* [24]. The significantly up-regulated *Vp*CypA2 mRNA expression level was observed in the hemocytes of *V. philippinarum* after the challenge with *L. anguillarum* [94]. The expression of CypA increased in the hepatopancreas of *M. rosenbergii* after *C. freundii* infection in the present study; the CypA mRNA expression level was significantly up-regulated in the hepatopancreas of *P. clarkii* after the challenge with *C. freundii* [14].

CTLs are key recognition proteins and play important roles in shrimp innate immunity for the recognition and clearance of pathogens [22,23,24,25]. *Pm*CL1 expression was significantly upregulated in the hepatopancreas and gill of *P. monodon* after the infection with *V. harveyi* and *V. anguillarum*, respectively [95]. The expressions of hepatopancreas-specific CTL (*Fc-hs*L) and CTLs (*Fc*lectin, *Fc*Lec3 and *Fc*Lec4) from *F. chinensis* were up-regulated following the challenge of shrimp with *V. anguillarum* or *S. aureus* [96,97,98,99]. In *L. vannamei*, the expressions of *Lv*CTL3 and *Lv*PLP (the perlucin-like protein, a typical CTL) were up-regulated after *V. parahaemolyticus* infection; two CTLs (*Lv*Lectin-1 and *Lv*Lectin-2) showed significantly increased expressions after the *L. anguillarum* challenge [100,101,102]. Two novel lectins (*Mn*CTLDcp2 and *Mn*CTLDcp3) from *M. nipponense* were up-regulated in the heart after the challenge with *A. hydrophila* [103]. The expression of CTL increased in the hepatopancreas of *M. rosenbergii* after *C. freundii* infection in the present study, and a significantly up-regulated CTL mRNA expression level was observed in the hepatopancreas of *P. clarkii* after the challenge with *C. freundii* [14].

In general, when *M. rosenbergii* was infected with *C. freundii*, CTL, as one of pattern-recognition receptor, immediately recognized the non-self material that had entered the prawn body. With *C. freundii* infection, enzymes involved in the antioxidant antibacterial system (SOD, CAT, GPx, GST, and MDA) were synthesized rapidly in the hepatopancreas and hemolymph, and the phosphatase enzymes (ACP and ALP) and LZM also actively participated in this immune response. In addition, the expressions of immune-related genes (Cu/Zn-SOD, CAT, GPx, GST, LZM, ACP, ALF, crustin, CypA, and CTL) in the hepatopancreas were significantly up- or down- regulated, which suggested that the immediate immune response system in *M. rosenbergii* offers defense against *C. freundii* infection. The results of this study indicated that all of these antioxidant enzymes and genes play important roles in the *M. rosenbergii* immune responses. Furthermore, the hepatopancreas are important immune tissues in the defense against *C. freundii*.

Antibiotics are natural or synthetic compounds and have become the main means for the prevention and treatment of infectious diseases due to their low cost, good curative effect, and simple operation [104,105]. Florfenicol, oxytetracycline, oxytetracycline hydrochloride, ormetoprim, and sulfamethazine were the five antibiotics approved by the FDA [106]. In the present study, florfenicol and ofloxacin were selected to evaluate their therapeutic effects against *C. freundii* in *M. rosenbergii*. Ofloxacin was forbidden in aquaculture and its use in the present study was just for scientific research. Florfenicol is a broad-spectrum and effective antibacterial, belonging to a part of the chloramphenicol family of drugs, that is widely used to control susceptible bacterial diseases in fish and shrimp farming [107,108,109,110,111]. Florfenicol and ofloxacin can stop bacteria from multiplying by interfering with bacterial protein production and DNA replication, respectively [31]. The increasd immune and antioxidant responses in *M. rosenbergii* after *C. freundii* challenge were results of *C. freundii* activated the host’s innate immune system. With antibiotics feeding, *C. freundii* in *M. rosenbergii* cannot multiplying well owing to the protein production or DNA replication interfering. Then, the innate immune system of *C. freundii* infected *M. rosenbergii* was more efficient against bacteria with the higher survival rate, immune and antioxidant responses. *C. freundii* can break the innate immune system of *M. rosenbergii* treated without antibiotics, damage the capital functional organs and cause death. Florfenicol was effective in the treatment of pseudotuberculosis in Yellowtail *Seriola quinqueradiata* [112], vibriosis in Goldfish *Carassius auratus* [113], and furunculosis in Atlantic Salmon *Salmo salar* [114], *V. anguillarum* infection in Atlantic cod *Gadus morhua* [106], vibriosis in Black Tiger Shrimps *P. monodon* [111], *V. harveyi* infection in *L. vannamei* [110]. Due to the adverse environmental consequences, alternatives to antibiotics have been developed, including probiotics, phage therapy, essential oils (EOs), and Chinese herbal medicine [31]. *Pseudomonas synxantha* and *Pseudomonas aeruginosa*-fed *Penaeus latisulcatus* showed a higher tolerance to the *V. harveyi* challenge [115]. Four phages that had lytic activity against *V. harveyi* were isolated, and were demonstrated to be effective in controlling the population of *V. harveyi* in hatchery systems and improved the survival of *P. monodon* [116]. The usage of EOs from *Cinnamosma fragrans* enhanced the survival of *P. monodon* larvae with *Vibrio penaeicidae* and *Vibrio splendidus* challenge and decreased bacterial concentration [117]. For the WBD in *M. rosenbergii*, we also conducted alternatives therapies, such as Chinese herbal medicine. We found strain GDZQ201912 was sensitive to *Terminalia chebula Retz*, *Scutellaria baicalensis Georgi*, *Caesalpinia sappan Linn*, *Rhus chinensis Mill*, and *Schisandra chinensis* as well as the evaluations of their therapies of WBD, survival rate, immune and antioxidant responses of *M. rosenbergii* (Unpublished data).

## 5. Conclusions

Overall, in the present study, the pathogenic bacterium *C. freundii* was isolated from WBD *M. rosenbergii* with a mass mortality, and the cause of WBD in *M. rosenbergii* was identified with the typical sign under the carapace. The TEM observation, physiological and biochemical characteristics, LD_50_, and antimicrobial susceptibility of the *C. freundii* isolate were studied. The therapeutic effects of antibiotics against WBD were also evaluated, and florfenicol was the most efficient antibiotic to defend against *C. freundii*. In addition, the results of the present study provide insights into the histopathological changes, antioxidant enzymatic activity, and changes in the expressions of immune-related genes of *M. rosenbergii* challenged with *C. freundii*, revealing the antibacterial immune responses in giant river prawn. These results will facilitate the development of therapies and management of the disease caused by this pathogen in aquaculture systems.

## Figures and Tables

**Table 1:** Ingredient composition and proximate analysis of commercial feed used for *M. rosenbergii*.

Ingredient	Dry Weight (%)
Rice bran	30.0
Soybean meal (44%)	25.0
Wheat midds	17.75
Menhaden meal (72%)	16.0
Meat, bone, and blood meal	11.0
Mineral premix ^a^	0.1
Vitamin premix ^b^	0.1
Choline	0.05
Proximate analysis (%)	
Moisture	12.3
Crude protein	36.2
Crude fat ^c^	6.3
Crude fiber	4.4
Ash	10.7
Nitrogen free extract (by difference)	30.1

^a^ Mineral premix contains: Mn, 11.0%; Zn, 10.0%; Fe, 8.0%; I, 0.36%; Co, 0.10%; Ca (carrier). ^b^ Vitamin premix contains: vitamin B1, 1.63%; riboflavin, 1.72%; pyridoxine, 1.2%; nicotinic acid, 9.97%; folie acid, 0.34%; vitamin B12, 0.003%; pantotbenic acid, 3.89%; ascorbic acid, 43.69%; vitamin A, 1700 IU/kg; vitamin D3 1200 IU/kg; vitamin E, 66.138 IU/kg; etboxyquin, 0.66%. ^c^ Acid hydrolysis.

**Table 2:** Primers used for quantitative real-time PCR (qRT-PCR).

Target Genes	Sequences (5′–3′)	Accessions
β-actin	Forward: GAGACCTTCAACACCCCCGC	AF221096.1
	Reverse: TAGGTGGTCTCGTGGATGCC	
ALF	Forward: ATCTGGCGTCGTTACCAAAAC	JQ364961.1
	Reverse: GAAATGAAACCTGATGATCGTC	
Crustin	Forward: AACGACTTCAAGTGCTTCGGGTCT	JQ413342.1
	Reverse: AAGCTTAGTGGTTTGCAGACGTGC	
CTL	Forward: ATGTTGACCTTAATGGCCAC	KX495215.1
	Reverse: CTTTTCTGTGGGCGTTTCTTC	
Cu/Zn-SOD	Forward: TCGCCTAACGAGGAGGTTC	DQ121374.1
	Reverse: CGGCTTCATCAGGATTTTGAG	
CypA	Forward: CTAATGCTGGACCCAACACC	EL696406.1
	Reverse: CCTCCACT CCAAT TCTAGCTGTAA	
LZM	Forward: TGCCATCAACCACCACAACT	AY257549.2
	Reverse: CCCCTTTCCCTTCCACTTCT	
CAT	Forward: AGCGAGATTGGCAAGAAGACACC	HQ668089.1
	Reverse: AAGGATGGTGACCTGGTGCGTGG	
GPx	Forward: TTCGCCCAGGGAACAATTT	EL696567.1
	Reverse: CCTTTTCACTGAGAATTACCCAG	
GST	Forward: GTTGTGCAGCATTGAGGTTTAT	HF570114.1
	Reverse: GTATCCTACACCATGTGCTCTG	
ACP	Forward: GTTTACACTCGCCTTATCCTCCG	JX975267.1
	Reverse: CTTTGTGCATGAACATGACCCTG	

**Table 3:** Morphological properties and conventional tests of strain GDZQ201912 isolated from *Macrobrachium rosenbergii*.

Characteristics	Strain GDZQ201912	*Citrobacter freundii* *
Gram reaction	−	−
Shape	Rod	Rod
Motility	+	+
Indole	−	V
Citrate	+	V
Methyl red	+	+
H_2_S	+	V
Ornithine	−	−
Malonate	−	V
Sucrose	+	+
Melibiose	+	+
Raffinose	+	V
Dulcitol	+	V
Adonitol	−	−

“+” positive reaction, “−” negative reaction; “V” variable (positive or negative reaction). * Reference strain data compiled from Bergey’s manual [38].

**Table 4:** Carbon source utilization reactions of strain GDZQ201912.

Characteristics	Strain GDZQ201912	*Citrobacter freundii* *
*cis*-Aconitate	+	+
*trans*-Aconitate	+	+
Adonitol	−	−
*L*-alanine	+	+
γ-Aminobutyrate	+	+
D-arabitol	−	−
Benzoate	−	−
Caprate	−	−
D-cellobiose	+	+
Dulcitol	+	+
D-galactose	+	+
*N*-acetyl-β-D-galactosamine	+	+
D-galacturonic acid	+	+
*L*-galactose	+	+
Esculin	−	−
D-fucose	+	+
D-glucose	+	+
Gentiobiose	+	+
Gentisate	+	+
*L*-glutamate	+	+
Glycerol	+	+
Inositol	+	+
*L*-lactate	+	+
Lactose	+	+
Lactulose	+	+
Maltitol	+	+
Melibiose	+	+
Phenylacetate	−	+
*L*-proline	+	+
Putrescine	−	+
Raffinose	+	+
D-sorbitol	+	+
Sucrose	+	+
D-turanose	−	−
*L*-tyrosine	−	+
Stachyose	−	−
D-fructose	−	−
*L*-fructose	+	+
D-mannose	+	+
*L*-rhamnose monohydrate	+	+
dextrin	−	−
D-glucosamic acid	+	+
D-salicin	+	+
*N*-acetylglucosamine	+	+
D-mannitol	+	+
*N*-acetylneuraminic acid	+	+
D-glucuronamide	−	−
D-serine	+	+
D-aspartic acid	+	+
*L*-arginine	−	−
Methyl pyruvate	+	+
Methyl D3phenyllactate	−	−
D-malic acid	+	+
*L*-malic acid	+	+

(+) positive and (−) negative. * Reference strain data compiled from Bergey’s manual [38].

**Table 5:** Antimicrobial susceptibility analysis of *C. freundii* isolates from *M. rosenbergii* (strain GDZQ201912).

Number	Antibiotics	Drug Content (µg/Pill)	Diameter of Inhibition Zone	Results
1	penicillin	10	6.00 ± 0.00	±
2	doxycycline	30	18.36 ± 0.16	+ +
3	florfenicol	30	28.57 ± 0.19	+ + +
4	norfloxacin	10	28.85 ± 0.21	+ + +
5	ofloxacin	5	29.91 ± 0.13	+ + +
6	amoxicillin	20	14.23 ± 0.11	+ +
7	ampicillin	10	6.00 ± 0.00	±
8	cefepime	30	41.12 ± 0.23	+ + +
9	ceftriaxone	30	36.78 ± 0.15	+ + +
10	neomycin	30	19.63 ± 0.12	+ + +
11	gentamicin	10	17.51 ± 0.18	+ + +
12	streptomycin	10	10.03 ± 0.10	+ +
13	clindamycin	2	6.00 ± 0.00	±
14	lincomycin	2	6.00 ± 0.00	±
15	tetracycline	30	26.56 ± 0.17	+ + +
16	novobiocin	30	6.00 ± 0.00	±
17	Polymyxin B	300	19.21 ± 0.14	+ + +
18	rifampin	5	10.11 ± 0.12	+ +

“+ + +”: Highly sensitive; “+ +”: Moderately sensitive; “±”: Drug resistance.

## Data Availability

The data presented in this study are available in the article.
